# Association between fusion and clinical outcomes after anterior cervical discectomy at 1-, 2- and 5-year follow-up

**DOI:** 10.1371/journal.pone.0337909

**Published:** 2025-12-15

**Authors:** Floor E. de Vries, Ignacio Mesina-Estarrón, Carmen L. A. Vleggeert-Lankamp

**Affiliations:** 1 Department of Neurosurgery, Leiden University Medical Center (LUMC), Leiden, the Netherlands; 2 Department of Neurosurgery, Spaarne Hospital, Haarlem/Hoofddorp, the Netherlands; 3 Computational Neuroscience Outcomes Center at Harvard, Department of Neurosurgery, Brigham and Women’s Hospital, Boston, United States of America; 4 Harvard Medical School, Boston, United States of America; Mayo Clinic Rochester: Mayo Clinic Minnesota, UNITED STATES OF AMERICA

## Abstract

**Introduction:**

Fusion achievement is considered a crucial factor in recovery following anterior discectomy. Nevertheless, the direct correlation between fusion and clinical outcomes, such as pain and disability, remains ambiguous due to inconsistent fusion measurement methods. Recent advancements in diagnostic fusion criteria now enable a more accurate fusion assessment. This study aimed to assess the association between fusion and clinical outcomes in patients undergoing anterior cervical discectomy.

**Methods:**

This post-hoc analysis was conducted using data from the NEtherlands Cervical Kinematics (NECK) trial (NTR1289). Patients with a single level herniated disc that underwent anterior cervical discectomy between 2010 and 2014 were evaluated at 52, 104, and 260 weeks. Fusion was assessed using dynamic radiographs, applying the de Vries-Vleggeert criterion (≤3.0° Cobb angle and ≤2.0 mm interspinous distance). Clinical outcomes included the Neck Disability Index (NDI) and Visual Analog Scale (VAS) for arm and neck pain. Linear and logistic regressions were performed to evaluate correlations.

**Results:**

Fusion was present in 57% (52 weeks), 75% (104 weeks), and 83% (260 weeks) of patients. Linear regression analyses revealed a clear trend suggesting favorable long-term VAS arm and neck pain scores in the patient’s demonstrating fusion with statistically significant lower VAS arm pain (mean difference: –18.9, 95% CI –36.9 to –0.9, p = 0.040) in the fusion group at 260 weeks follow-up. At earlier follow-up points, the differences in VAS arm pain did show a trend, but did not reach statistical significance (W52: 6.0, 95% CI –6.6 to 18.6, p = 0.346; W104: –11.5, 95% CI –24.2 to 1.3, p = 0.076). VAS neck pain scores showed a trend, but no statistically significant differences between groups across follow-up (W52: 3.2, 95% CI –8.1 to 14.5, p = 0.572; W104: –1.5, 95% CI –13.8 to 10.8, p = 0.808; W260: –12.7, 95% CI –30.9 to 5.6, p = 0.170). No significant differences were observed in NDI outcomes at any time point (W52: 3.2, 95% CI –4.8 to 11.2, p = 0.431; W104: –3.7, 95% CI –11.6 to 4.2, p = 0.358; W260: 2.5, 95% CI –7.2 to 12.1, p = 0.608). Logistic regression analysis using success rates based on established cut-off values showed a trend towards patients with fusion having markedly higher odds of success long term, with a significant higher odd of success in VAS arm pain at 260 weeks FU (OR 9.88, 95% CI 1.55–62.80, p = 0.015).

**Conclusion:**

A comparative analysis indicated reduced arm and neck pain in the fusion group at the 260-week follow-up. This finding becomes apparent only in the long-term post-intervention period, suggesting that muscle tension may function as a natural brace during the initial years following surgery. This tension effectively limits excessive flexion-extension movements, thereby mitigating discomfort. These results have potential implications for routine clinical surgical practice. Future studies with larger sample sizes are needed to validate these findings, including short-term follow-up.

## Introduction

Cervical radicular syndrome (CRS) is commonly caused by compromising a cervical nerve root due to a cervical disc herniation [1,2]. Anterior cervical discectomy is a widely performed surgical procedure for this condition, wherein the herniated disc is excised and the nerve root decompressed via an anterior approach [3–5]. Following disc removal, an implant can be placed between the vertebral bodies to maintain foraminal height, and to induce bony fusion [6–8].

Fusion between the adjacent vertebrae is considered an important factor for successful recovery following anterior discectomy [9]. An anterior discectomy can temporarily compromise the stability of the cervical spine after surgery, potentially resulting in unbalanced pressure on the facet joints and uncovertebral joints, and in misalignment, which may lead to neck pain and disability [10]. Therefore, patients’ daily activities are temporarily restricted until stability and/or bony fusion is deemed to have been achieved [7,11].

Knowledge on the actual correlation between fusion and clinical outcome is however unclear. Although several clinical and radiological studies have explored the occurrence of bony fusion, as well as the clinical outcomes of patients undergoing anterior cervical discectomy, few studies have examined the correlation between fusion and clinical outcomes [12–17]. As a standardized method for assessing fusion has been lacking, the interpretation and comparison of these results have been challenging, and outcomes are varying. Recently, a diagnostic criterion for assessing fusion has been established [18], allowing for proper establishment of the association between fusion and clinical outcome.

This new method allowed us to apply the standardized fusion criterion to previously collected clinical data from patients that underwent anterior discectomy who were randomized to variating intervertebral devices [19]. All patient groups demonstrated clinical improvement after surgery, regardless of the treatment received. However, on an individual basis, not all patients achieved significant recovery and some even required a reoperation. Adjacent segment degeneration (ASD) accounted only partially for disappointing outcomes; the number of ASD-related reoperations after five years was low, with three cases each in the arthroplasty and cage groups. Besides that, patients undergoing discectomy alone demonstrated less favorable outcomes across several measures, including NDI, EQ-5D, and perceived recovery of arm pain [19].

The current study aimed to assess the association between fusion and clinical outcomes in this group of patients, to evaluate (lack of) fusion as a determinator for treatment success.

## Materials and methods

### Patients

A post-hoc analysis was performed on data collected as part of the NEtherlands Cervical Kinematics (NECK) trial [19,20], a prospective, double-blinded multicenter randomized clinical trial in which patients with cervical radiculopathy due to a cervical disc herniation were randomly subjected to anterior cervical discectomy with arthroplasty (ACDA), anterior cervical discectomy and fusion (ACDF) or anterior cervical discectomy without an intervertebral device (ACD) between 26-10-2010 and 10-07-2014. All patients underwent single-level surgery. The Central Medical Ethics Committee of the Leiden University Medical Center, and the boards of directors of the involved hospitals granted approval for the trial (Dutch Trial Register Number: NTR1289). Written informed consent was obtained from all patients. A total of 109 patients were initially included in the NECK trial. Patients (aged 18–65 years old) with radicular signs and symptoms in the arm (pain, paresthesia or paresis in a specific nerve root distribution) for at least 8 weeks and for whom conservative therapy failed were eligible for inclusion. Patients with previous cervical surgery (either anterior or posterior), absence of motion, increased anteroposterior translation, very narrow (<3 mm) intervertebral space, severe segmental kyphosis (>3°) at the index level on static or dynamic X-rays, neck pain only and symptoms and signs of myelopathy were excluded. Furthermore, patients with metabolic and bone diseases (osteoporosis, severe osteopenia), neoplasm or trauma of the cervical spine, spinal anomaly (Klippel Feil, Bechterew, OPLL), severe mental or psychiatric disorders were excluded [19]. Patients underwent either ACDA with an activC^®^ prosthesis, ACDF with an interbody PEEK cage filled with synthetic bone substitute or autologous bone (chips locally harvested), or ACD without an intervertebral device. Patients were followed up to 5 years after surgery.

### Radiographic assessment

**Fig 1:**
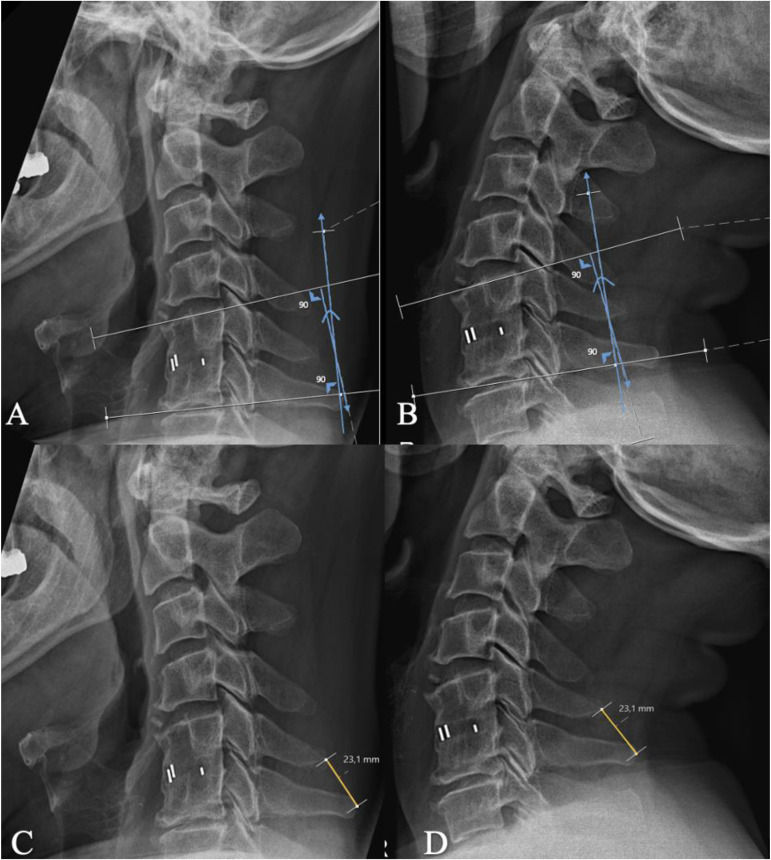
Examples of the quantitative measurements. ^*^ Blue lines: Measurement [1] of the Cobb angle of the operated level on flexion **(A)** and extension **(B)** Yellow lines: Measurement [2] of the interspinous distance (ISD) of the operated level on flexion **(C)** and extension **(D)**. ^*^This figure was published in “Assessing accuracy of measurement methods for bony fusion assessment after anterior cervical discectomy”, de Vries et al., The Spine Journal, Volume 24, Issue 11, 2024, Pages 2035–2044, Copyright Elsevier (2025).

The presence or absence of fusion was the primary independent variable of interest in our analyses. Flexion and extension radiographs were obtained at 52-, 104- and 260-weeks follow-up (FU). To determine the fusion at the target level, two measurements were performed at all three timepoints on flexion and extension X-rays (Fig 1): 1) the Cobb angles of the operated level were measured as the angle between the line drawn parallel to the rostral endplate of the upper vertebral body and the line drawn parallel to the caudal endplate of the lower vertebral body. The difference between the angle in flexion and the angle in extension was calculated; 2) the distances between the distal ends of the spinous processes adjacent to the operated level were measured. The difference between the distance in flexion and in extension was calculated (Interspinous Distance; ISD). Two investigators independently performed these measurements, both using the same measurement program (PACS Sectra IDS7). The two investigators were trained to perform these measurements by a senior spine surgeon. Both investigators were blinded to the other investigator’s measurements. Successful fusion was achieved when the cut-off values were ≤ 3.0° for Cobb angle and ≤ 2.0 mm for interspinous distance, according to the de Vries-Vleggeert criterion [18].

### Clinical outcome assessment

The Neck Disability Index (NDI) evaluates functionality and pain of the neck and upper limb. The NDI is a 10-item questionnaire on three different aspects: pain intensity, daily work-related activities and non-work-related activities. Each item is scored from 0 to 5 and the total score ranges from 0 (best score) to 50 (worst score). This 50-point score was converted to a 100-point scale (50 points = 100 points). The NDI is a modification of the Oswestry Low Back Pain Index and has been shown to be reliable and valid for patients with cervical pathology [21–23].

The Visual Analogue Scale for arm pain (VAS arm) and for neck pain (VAS neck) measures the experienced pain intensity during the week before evaluation. Pain was assessed on a horizontal 100 mm scale varying from 0 mm (no pain) to 100 mm (worst pain imaginable). Patients did not see the results of earlier assessments. Reliability, validity and responsiveness of VAS have been shown previously [24].

These outcome scores were obtained at 52-, 104- and 260-weeks FU.

To additionally investigate the association between fusion and clinical outcomes for the individual patients, rather than for the mean values, we utilize cut-off values to differentiate between “success” and “non-success” after surgery for cervical radiculopathy based on criteria established by Mjaset et al. [25] to determine outcome classifications for the individual patients.

### Statistical analysis

Demographic data are presented as the mean (±standard deviation (sd)) for continuous variables and as counts for categorical variables. Differences in demographic characteristics by fusion status were assessed using two-sample t-tests or chi-squared tests. For the difference in ISD and the difference in Cobb angle on the flexion-extension images, interobserver variance was calculated using the Intraclass Correlation Coefficient (ICC) (0–0.20 [slight agreement], 0.21–0.40 [fair agreement], 0.41–0.60 [moderate], 0.61–0.80 [substantial agreement], 0.81–1.00 [almost perfect agreement]) [26]. A linear regression analysis was performed for the correlation between fusion and clinical outcomes, adjusted for clinical scores at baseline, and additionally for sex, age and intervertebral device (ACDA, ACDF or ACD) at all three timepoints: 52, 104 and 260 weeks after randomization. A logistic regression analysis was conducted for analysis of the dichotomized clinical data by the cut-off values for success criteria after surgery for cervical radiculopathy established by Mjaset et al. [25] Mjaset et al. defined percentage change scores to differentiate between success and non-success for the NDI (35.1%), VAS arm (47.2%) and VAS neck (38.8%). Values of *P* < 0.05 were considered statistically significant. All statistics were performed using IBM SPSS software, version 29.0.

## Results

### Patient demographics

**Fig 2:**
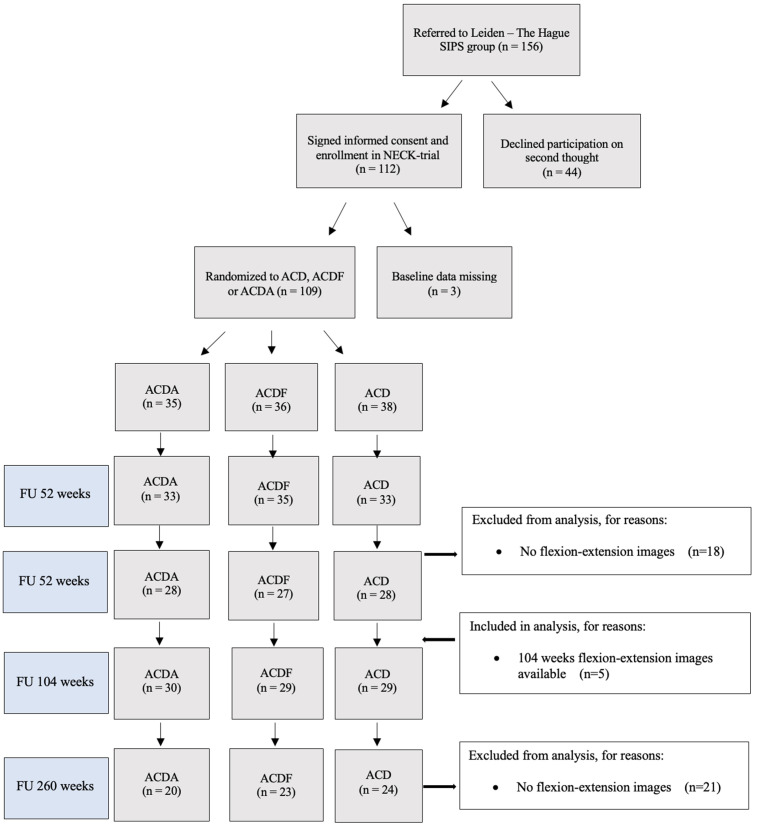
Flowchart of participants. *ACDA = anterior cervical discectomy with arthroplasty, ACDF = anterior cervical discectomy and fusion, ACD = anterior cervical discectomy without an intervertebral device.

109 patients were included in the NECK trial. No differences were noted in patient sex, age, and baseline NDI and VAS pain scores [20]. At five-years FU, data was available for 89 out of 109 patients (Fig 2). Only the clinical FU data of patients who returned for a flexion-deflexion X-ray were included in the analysis. There were no significant differences between baseline data of patients with and without radiological FU (S1 Appendix).

### Fusion status

The Intraclass correlation coefficients between the investigators was 0.95 (95% CI: 0.91–0.97) for the Cobb angle measurements and 0.95 (95% CI: 0.90–0.97) for the ISD measurements, showing excellent agreement.

At 52 weeks FU, 83 patients had a flexion-deflexion image available and 57% of these demonstrated fusion. At 104 weeks, 88 patients had appropriate imaging available and 75% of these demonstrated fusion. Five years after inclusion 67 patients returned for a flexion-deflexion X-ray and 83% of them demonstrated fusion.

Demographics for the patients exhibiting fusion vs. non-fusion at 52-, 104- and 260-weeks FU is presented in Table 1.

**Table 1:** Participants Demographics and Characteristics based on the distribution fusion vs non-fusion at 52-, 104- and 260-weeks FU.

Variable | 52 weeks	Fusion (N = 47)	Non-fusion (N = 36)	P-value
Male: Female	20: 27	10: 16	0.240
Mean age*	48.6 ± 7.7	46.1 ± 8.0	0.150
C5-6: C6-7	26: 21	20: 16	0.823
ACDA: ACDF: ACD	8: 17: 22	20: 10: 6	< 0.001
Baseline NDI	40.0 ± 15.4	46.1 ± 16.6	0.092
Baseline VAS arm	55.9 ± 22.3	61.0 ± 22.1	0.305
Baseline VAS neck	49.3 ± 27.2	53.0 ± 27.1	0.540
**Variable | 104 weeks**	**Fusion (N = 66)**	**Non-fusion (N = 22)**	**P-value**
Male: Female	28: 38	8: 14	0.617
Mean age*	47.9 ± 7.8	46.9 ± 9.4	0.623
C5-6: C6-7	32: 34	14: 8	0.218
ACDA: ACDF: ACD	15: 25: 26	14: 4: 4	0.002
Baseline NDI	41.4 ± 14.4	49.0 ± 17.9	0.053
Baseline VAS arm	56.7 ± 22.5	70.1 ± 16.0	0.002
Baseline VAS neck	47.7 ± 26.9	57.6 ± 26.1	0.136
**Variable | 260 weeks**	Fusion** (N = 56)	**Non-fusion (N = 11)**	**P-value**
Male: Female	23: 33	3: 8	0.508
Mean age*	48.9 ± 6.8	51.5 ± 9.8	0.289
C5-6: C6-7	30: 26	8: 3	0.326
ACDA: ACDF: ACD	13: 20: 23	7: 3: 1	< 0.001
Baseline NDI	40.8 ± 14.8	47.6 ± 18.7	0.205
Baseline VAS arm	54.3 ± 22.4	68.6 ± 22.4	0.058
Baseline VAS neck	49.2 ± 27.5	63.3 ± 23.2	0.119

ACDA = anterior cervical discectomy with arthroplasty, ACDF = anterior cervical discectomy and fusion, ACD = anterior cervical discectomy without an intervertebral device, NDI = neck disability index, VAS = visual analog scale.

*At time of surgery (mean ± sd)

### Clinical outcomes

#### NDI.

**Fig 3:**
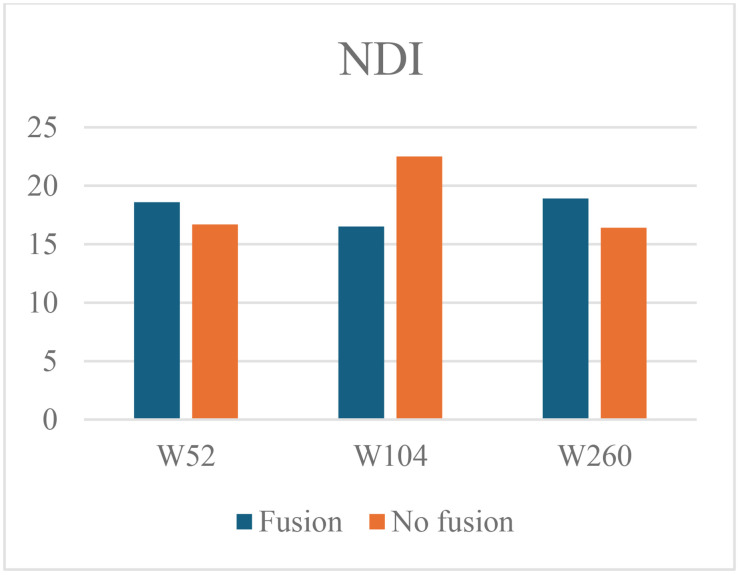
Mean NDI values for the fusion and non-fusion group at 52-, 104- and 260 weeks follow-up.

At 52 weeks FU the mean NDI was 18.6 ± 15.7 in the patients with fusion and 16.7 ± 18.9 in the patients without fusion (mean difference 1.9, 95% CI –5.9 to 9.7, p = 0.627). At 104 weeks the mean NDI was 16.5 ± 14.4 in the fusion group and 22.5 ± 22.1 in the non-fusion group (mean difference −6.0, 95% CI –16.6 to 4.6, p = 0.252). However, at 260 weeks values became more equivalent again: the mean NDI was 18.9 ± 18.1 in the patients with fusion and 16.4 ± 15.8 in the patients without fusion (mean difference 2.5, 95% CI –9.2 to 14.2,p = 0.670) (Fig 3).

At 52 weeks, the linear regression model (additionally adjusted for sex, age and intervertebral device) shows a slightly higher disability score when fusion is present (B 3.170, 95% CI –4.82 to 11.16, p = 0.431). At 104 weeks, there is a lower disability score when fusion is achieved (B −3.664, 95% CI –11.56 to 4.23, p = 0.358). At 260 weeks, the disability score again slightly increases (B 2.485, 95% CI –7.17 to 12.14, p = 0.608). Thus, five years after surgery the NDI is on average 2.5 points (on a 100-point scale) higher in patients that demonstrate fusion at that time, and statistical significance of these results is lacking (Table 2).

**Table 2:** Linear regression analysis for fusion vs non-fusion and NDI, VAS arm pain and VAS neck pain as outcome.

NDI						
FU	B^a^	(95% CI)^a^	*p* ^a^	B^b^	95% CI^b^	*p* ^b^
W52	4.183	-2.767 – 11.133	0.234	3.170	-4.815 – 11.156	0.431
W104	-2.644	-10.198 – 4.909	0.488	-3.664	-11.556 – 4.227	0.358
W260	7.418	-2.450 – 17.286	0.138	2.485	-7.171 – 12.142	0.608
^a^ Model is adjusted for NDI at baseline						
^b^ Model is additionally adjusted for sex, age and intervertebral device						
CI = Confidence Interval						
**VAS arm**						
	**B** ^**a**^	**(95% CI)** ^**a**^	***p*** ^**a**^	**B** ^**b**^	**95% CI** ^**b**^	***p*** ^**b**^
W52	5.499	-5.652 – 16.649	0.329	6.011	-6.609 – 18.631	0.346
W104	-7.541	-19.452 – 4.369	0.211	-11.491	-24.232 – 1.250	0.076
W260	-11.395	-28.687 – 5.897	0.193	-18.903	-36.934 – -0.872	**0.040**
^a^ Model is adjusted for VAS arm pain at baseline						
^b^ Model is additionally adjusted for sex, age and intervertebral device						
CI = Confidence Interval						
**VAS neck**						
	**B** ^**a**^	**(95% CI)** ^**a**^	***p*** ^**a**^	**B** ^**b**^	**(95% CI)** ^**b**^	***p*** ^**b**^
W52	3.071	-6.950 – 13.091	0.544	3.221	-8.070 – 14.512	0.572
W104	-0.600	-11.926 – 10.727	0.916	-1.509	-13.824 – 10.805	0.808
W260	-4.259	-21.960 – 13.443	0.632	-12.675	-30.925 – 5.576	0.170
^a^ Model is adjusted for VAS neck pain at baseline						
^b^ Model is additionally adjusted for sex, age and intervertebral device						
CI = Confidence Interval						

Overall, both the visual data and statistical models indicate that fusion does not significantly impact NDI outcomes.

#### VAS arm pain.

**Fig 4:**
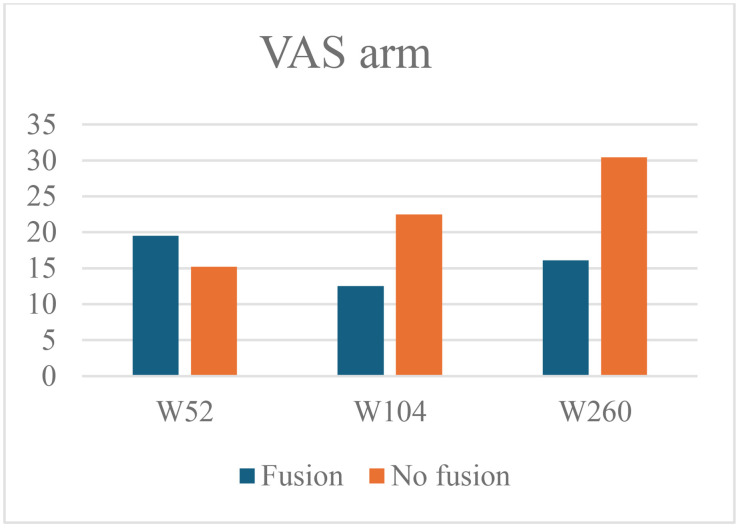
Mean VAS arm pain scores in the fusion and non-fusion group at 52-, 104- and 260 weeks follow-up.

At 52 weeks, the mean VAS arm score was 19.5 ± 27.0 in the fusion group and 15.2 ± 23.5 in the non-fusion group (mean difference 4.3, 95% CI –7.0 to 15.5, p = 0.451). At 104 weeks, the mean VAS arm score decreased to 12.5 ± 20.2 in the fusion group and increased to 22.5 ± 31.6 in the non-fusion group (mean difference −10.0, 95% CI –24.8 to 4.7, p = 0.174). At 260 weeks, the mean VAS arm score was 16.1 ± 25.0 in the fusion group and 30.4 ± 27.1 in the non-fusion group (mean difference −14.3, 95% CI –31.0 to 2.5, p = 0.094) (Fig 4).

At 52 weeks, the adjusted linear regression model shows slightly more arm pain when fusion is present (B 6.011, 95% CI –6.61 to 18.63, p = 0.346). At 104 weeks, there is a lower VAS arm pain score when fusion is achieved (B −11.491, 95% CI –24.23 to 1.25, p = 0.076). A statistically significant lower VAS arm pain score is displayed in patients demonstrating fusion at 260 weeks (B −18.903, 95% CI –36.93 to –0.87 p = 0.040). Five years after surgery the arm pain is on average 18.9 mm (on a 100 mm scale) lower in the patients that demonstrate fusion at that time (Table 2).

#### VAS neck pain.

**Fig 5:**
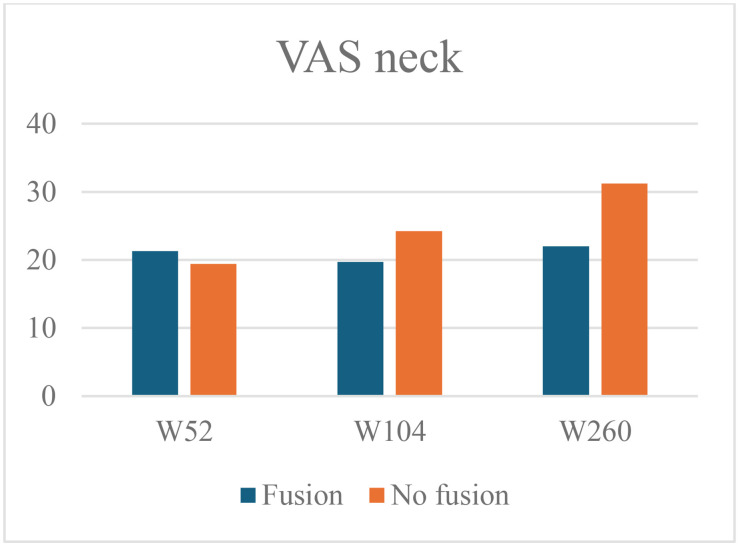
Mean VAS neck pain scores in the fusion and non-fusion group at 52-, 104- and 260 weeks follow-up.

At 52 weeks, the mean VAS neck pain was 21.3 ± 24.2 in the fusion group and 19.4 ± 24.1 in the non-fusion group (mean difference 1.9, 95% CI –8.8 to 12.5, p = 0.727). At 104 weeks, the mean VAS neck pain was 19.7 ± 22.5 in the fusion group and 24.2 ± 32.1 in the non-fusion group (mean difference –4.5, 95% CI –19.6 to 10.6, p = 0.547). At 260 weeks, the mean VAS neck score was 22.0 ± 26.2 in the fusion group and increased to 31.2 ± 34.1 in the non-fusion group (mean difference −9.2, 95% CI –27.4 to 9.0, p = 0.318) (Fig 5).

At 52 weeks, the adjusted linear regression model indicates slightly more neck pain when fusion is present (B 3.221, 95% CI –8.07 to 14.51, p = 0.572). At 104 the VAS neck pain scores are slightly lower with fusion present (B −1.509, 95% CI –13.82 to 10.81, p = 0.808). At 260 weeks, the VAS neck pain decreases further in patients demonstrating fusion (B −12.675, 95% CI –30.93 to 5.58, p = 0.170). Thus, five years after surgery the VAS neck pain score is on average 12.7 mm (on a 100 mm scale) lower in the patients that demonstrate fusion at that time. While a trend can be observed, the results show no statistically significant differences in VAS neck pain between the fusion and non-fusion groups (Table 2).

#### Cut-off values by Mjaset et al. [25].

The logistic regression model adjusted for sex, age, intervertebral device and clinical score at baseline, using success rates defined according to cut-off values established by Mjaset et al. [25], showed no significant associations between fusion and NDI outcomes at any follow-up point. At 52 weeks, the odds ratio (OR) for success in patients with fusion compared to those without was 0.60 (95% CI 0.17–2.15, p = 0.430). At 104 weeks, the OR increased slightly to 1.67 (95% CI 0.40–7.08, p = 0.485), and at 260 weeks the OR was 0.72 (95% CI 0.05–9.92, p = 0.804) (Table 3).

**Table 3:** Logistic regression analysis for fusion vs non-fusion and NDI, VAS arm pain and VAS neck pain using cut-off values established by Mjaset et al. [25].

NDI						
FU	Exp(B)^a^	(95% CI)^a^	*p* ^a^	Exp(B)^b^	95% CI^b^	*p* ^b^
W52	0.506	0.164 – 1.564	0.237	0.597	0.165 – 2.153	0.430
W104	1.202	0.339 – 4.265	0.776	1.672	0.395 – 7.079	0.485
W260	0.162	0.016 – 1.642	0.123	0.717	0.052 – 9.921	0.804
^a^ Model is adjusted for differences of NDI at baseline						
^b^ Model is additionally adjusted for sex, age and intervertebral device						
CI = Confidence Interval						
**VAS arm**						
	**Exp(B)** ^**a**^	**(95% CI)** ^**a**^	***p*** ^**a**^	**Exp(B)** ^**b**^	**95% CI** ^**b**^	***p*** ^**b**^
W52	0.508	0.170 – 1.517	0.225	0.388	0.104 – 1.448	0.159
W104	2.202	0.650 – 7.451	0.205	3.469	0.876 – 13.736	0.076
W260	3.877	0.923 – 16.283	0.064	9.880	1.554 – 62.800	**0.015**
^a^ Model is adjusted for differences of VAS arm pain at baseline						
^b^ Model is additionally adjusted for sex, age and intervertebral device						
CI = Confidence Interval						
**VAS neck**						
	**Exp(B)** ^**a**^	**(95% CI)** ^**a**^	***p*** ^**a**^	**Exp(B)** ^**b**^	**(95% CI)** ^**b**^	***p*** ^**b**^
W52	0.753	0.279 – 2.033	0.576	0.633	0.201– 2.530	0.601
W104	0.817	-0.255– 2.613	0.733	0.713	-13.824 – 10.805	0.808
W260	1.437	0.336 – 6.142	0.625	2.367	0.457 – 12.271	0.305
^a^ Model is adjusted for differences of VAS neck pain at baseline						
^b^ Model is additionally adjusted for sex, age and intervertebral device						
CI = Confidence Interval						

For the VAS arm pain the additionally adjusted OR for success at 52 weeks was 0.39 (95% CI 0.10–1.45, p = 0.159). At 104 weeks, patients with fusion showed higher odds of achieving success compared with those without (OR 3.47, 95% CI 0.88–13.74, p = 0.076). At 260 weeks, the higher odds of achieving success in the fusion group became statistically significant (OR 9.88, 95% CI 1.55–62.80, p = 0.015) (Table 3).

For VAS neck pain, no statistically significant associations were observed across follow-up. At 52 weeks, the OR was 0.63 (95% CI 0.20–2.53, p = 0.601). At 104 weeks, the OR increased to 0.71 (95% CI −13.82–10.81, p = 0.808). At 260 weeks, the odds of success were 2.37 times higher in patients with fusion compared to those without (OR 2.37, 95% CI 0.46–12.27, p = 0.305), indicating a trend toward improved long-term VAS neck outcomes in the fusion group, albeit without statistical significance.

## Discussion

Achieving fusion after anterior cervical discectomy is generally considered a desired outcome. The impact of non-fusion on clinical outcome however, remains debatable and proper research on this topic is scarce [27]. This study investigated the association between the occurrence of fusion and neck disability and arm- and neck pain during the five years following anterior discectomy for a herniated disc causing cervical radiculopathy. Although statistical significance was only reached for VAS arm pain at 260 weeks follow-up, a clear trend was discerned that arm and neck pain were more favorable in patients demonstrating fusion.

Previous studies have found conflicting results on the correlation between fusion and clinical outcomes. Klingler et al. [14] reviewed fusion status in 107 patients that underwent ACDF with 3 different cages (PEEK, Sulcem PMMA spacers or Palacos PMMA spacers) and reported that clinical outcomes are generally not significantly correlated with fusion status. Patients were determined as fused in that study if continuous trabecular bone bridges through or around the implant were clearly present on CT scans. This assessment is based on expert opinion, making the fusion status less reliable. Using this criterion, and correlating it to clinical outcome (VAS, NDI, and SF-36 physical and mental component) revealed that, only in the Sulcem PMMA group, fused patients demonstrated a statistically significant improvement in the physical component summary of the SF-36 compared to non-fused patients (P = 0.024). Fused patients across all substitute groups exhibited less pain and better functionality than the non-fused patients, although these differences did not reach statistical significance.

Shiban et al. [12] studied long-term fusion outcomes after ACDF (1-, 2- and 3-level surgery) in 318 patients. In agreement with our method, Shiban et al. evaluated stability rather than bony fusion, since they defined the measurement of interspinous distance on dynamic radiographs of ≥ 2 mm as non-fusion. This cut-off value corresponds with the De Vries-Vleggeert criteria [18] and their results align with ours: they demonstrated that non-fusion was correlated with higher VAS pain levels, which supports our trend findings that patients with fusion experience lower VAS arm and neck pain.

Noordhoek et al. [7] performed a systematic review on fusion after ACDF and reviewed 18 studies that examined the correlation between fusion accomplishment and clinical outcomes. The methods used to evaluate fusion were mainly qualitative and varied from scoring trabeculae on CT or X-ray to evaluating dynamic X-rays. Only three studies reported a correlation between the absence of fusion and poorer clinical outcomes (1 study CT trabeculae, 1 study X-ray trabeculae, 1 study dynamic X-ray), while 15 studies found no statistically significant correlation. The studies that did observe a correlation tended to have lower fusion rates compared to those that did not. This suggests that the studies that report a relatively low rate of non-fusion use non-reliable fusion assessment methods, leading to a disbalance in fusion outcome groups. This limits the ability to perform meaningful statistical analysis, rendering the results non-significant.

An updated systematic review [27] tried to examine the association between fusion and clinical outcomes and included only studies that used a quantitative method (Cobb angle or interspinous distance measurements) to score fusion. Moreover, the correlation between clinical outcomes and the achievement of fusion was evaluated at different time points after surgery. It was concluded that outcomes on the relation between bony fusion and clinical outcomes are inconsistent, indicating a need for further research focusing on this aspect to better understand and improve patient care post-ACDF.

It is remarkable that the trend for more favorable clinical outcome becomes apparent only after five years of follow up, whereas the one- and two-year follow-up data are not convincing. In a previous publication, wherein we compared the clinical outcomes of three different surgical techniques, we also observed a significant difference between the groups only after five years, not after one and two years [19]. It was shown that in patients that underwent discectomy without the placement of an intervertebral device (ACD) performed worse at that time, and we hypothesized that this was due to absence of fusion. In the current analysis, did indeed demonstrate that the absence of fusion is associated with a trend towards worse clinical outcomes. However, we adjusted for treatment, and the factor ‘surgical technique’ was not significantly influencing clinical outcome. Moreover, 96% of the patients that underwent surgery without an intervertebral device demonstrated fusion (S2–S3 Appendix). In conclusion, the worse clinical outcome in ACD patients cannot be explained by absence of fusion. This highlights the need for further research to investigate the reasons for poorer outcomes in ACD and may involve factors related to impaired sagittal balance.

The trend that better clinical outcome in patients that demonstrated fusion may be explained by the hypothesis that ongoing (micro)mobility leads to neck pain. Apparently, this only becomes evident long-term post-intervention, suggesting that muscle tension acts as a natural brace during the first years after surgery, effectively restricting excessive flexion-extension and preventing associated discomfort.

Rather than finding explanations for the association between fusion and clinical outcome, it must be considered that fusion may not have a meaningful impact on clinical outcomes in most patients. Fusion does seem to matter, but the data could be obscured by multiple other variables. We did account for a few covariates in the linear regression, but it could be that other factors play an additional role in long-term clinical follow-up. Nevertheless, it is essential to further investigate the association between fusion and clinical outcome in larger studies, as it may impact daily clinical surgical practice.

This study has several limitations that may have affected the validity and generalizability of its findings. First, as this is a post-hoc analysis of prospectively collected data, the original intent of the performed study was not to analyze the influence of fusion on clinical outcomes and therefore was not powered to perform these analyses. The small sample size reduces the statistical power of the study, making it more difficult to detect meaningful differences or associations in clinical outcomes between the fusion and non-fusion group. Another limitation is the prosthesis implant in one third of patients. The prosthesis is designed to give mobility in the cervical spine, and this may result in another type of mobility than mobility in non-fusion status after this was intended via ACD or ACDF. We corrected for this in the linear regression analysis, but some bias by factors unknown to us, cannot be ruled out. Moreover, the de Vries-Vleggeert criterion is based on the absence of movement and thus rather tests stability than bony fusion. Lastly, the study did not account for potential additional confounding variables such as body mass index (BMI) or smoking, which could have influenced the outcomes.

Notwithstanding, this study had several strengths. As a gold standard for fusion assessment has been lacking, previous studies have used varying methods to determine fusion, often relying on CT scans evaluated as “expert opinion” or using different cutoff values. A diagnostic criterion was introduced by us to reliably determine fusion, which adds to the validity of the results. Furthermore, we reviewed differences in clinical outcomes between fusion and non-fusion groups at different time points.

## Conclusion

This study investigated the influence of fusion after anterior discectomy on clinical outcomes. The findings reveal a significant association between stability and long-term outcome of VAS arm pain and a trend between stability and long-term outcome of VAS neck pain. It is conceivable that other sources of persistent or developing discomfort after anterior discectomy are more determinant for outcome than fusion or stability. This study underscores the need for further research including a larger sample size, using the de Vries-Vleggeert diagnostic criterion.

## Supporting information

S1 Appendix Characteristics of patients with clinical FU and radiological FU. (DOCX)

S2 Appendix Linear regression analysis for fusion vs non-fusion and NDI, VAS arm pain and VAS neck pain as outcome, without ACDA patients (only ACD and ACDF). (DOCX)

S3 Appendix Clinical and radiological outcomes for each intervertebral device at 260 weeks FU. (DOCX)
