# Supplementary material for: Association between fusion and clinical outcomes after anterior cervical discectomy at 1-, 2- and 5-year follow-up
Source: PLoS One. 2025 Dec 15;20(12):e0337909. doi: 10.1371/journal.pone.0337909 (PMC12704901; doi:10.1371/journal.pone.0337909)
Supplement: S1 Appendix — (DOCX) [file pone.0337909.s001.docx]

**Supporting information**

**S1 Appendix.** Characteristics of patients with clinical FU and radiological FU

1. *52 weeks FU*

| **Variable** | **Clinical FU (n=94)** | **Radiological FU (n=83)** | **P-value** |
| --- | --- | --- | --- |
| Male : Female | 42 : 52 | 43 : 40 | 0.640 |
| Mean age* | 47.4 ± 8.1 | 47.5 ± 7.9 | 0.912 |
| ACDA : ACDF : ACD | 32 : 31 : 32 | 28 : 27 : 28 | 0.994 |
| NDI | 17.7 ± 16.3 | 17.8 ± 17.0 | 0.967 |
| VAS arm | 17.0 ± 24.7 | 17.6 ± 25.5 | 0.861 |
| VAS neck | 20.4 ± 23.6 | 20.5 ± 24.1 | 0.984 |

1. *104 weeks FU*

| **Variable** | **Clinical FU (n=91)** | **Radiological FU (n=88)** | **P-value** |
| --- | --- | --- | --- |
| Male : Female | 38 : 53 | 36 : 52 | 0.908 |
| Mean age* | 47.5 ± 8.1 | 47.6 ± 8.2 | 0.951 |
| ACDA : ACDF : ACD | 31 : 30 : 30 | 29 : 29 : 30 | 0.983 |
| NDI | 18.1 ± 17.3 | 17.9 ± 16.6 | 0.937 |
| VAS arm | 15.4 ± 24.5 | 15.0 ± 23.8 | 0.915 |
| VAS neck | 21.2 ± 26.0 | 20.9 ± 25.2 | 0.923 |

1. *260 weeks FU*

| **Variable** | **Clinical FU (n=74)** | **Radiological FU (n=67)** | **P-value** |
| --- | --- | --- | --- |
| Male : Female | 29 : 45 | 26 : 41 | 0.963 |
| Mean age* | 48.6 ± 7.6 | 49.3 ± 7.4 | 0.565 |
| ACDA : ACDF : ACD | 25 : 25 : 24 | 20 : 23 : 24 | 0.864 |
| NDI | 18.0 ± 17.4 | 18.5 ± 17.6 | 0.886 |
| VAS arm | 22.8 ± 27.3 | 23.5 ± 27.6 | 0.868 |
| VAS neck | 18.3 ± 25.8 | 18.5 ± 25.7 | 0.972 |

ACDA = anterior cervical discectomy with arthroplasty, ACDF = anterior cervical discectomy and fusion, ACD = anterior cervical discectomy without an intervertebral device, NDI = neck disability index, VAS = visual analog scale.

*At time of surgery (mean ± sd)
