# Supplementary material for: Association between fusion and clinical outcomes after anterior cervical discectomy at 1-, 2- and 5-year follow-up
Source: PLoS One. 2025 Dec 15;20(12):e0337909. doi: 10.1371/journal.pone.0337909 (PMC12704901; doi:10.1371/journal.pone.0337909)
Supplement: S2 Appendix — (DOCX) [file pone.0337909.s002.docx]

**Supporting information**

**S2 Appendix.** Linear regression analysis for fusion vs non-fusion and NDI, VAS arm pain and VAS neck pain as outcome, without ACDA patients (only ACD and ACDF).

**NDI**

| **FU** | **B^a^** | **(95% CI)^a^** | ***p*^a^** | **B^b^** | **95% CI^b^** | ***p*^b^** |
| --- | --- | --- | --- | --- | --- | --- |
| W52 | 8.708 | 0.529 – 16.886 | 0.037 | 9.944 | 1.049 – 18.839 | 0.029 |
| W104 | -4.482 | -15.355 – 6.392 | 0.412 | 2.218 | -12.465 – 16.901 | 0.762 |
| W260 | 5.129 | -10.145 – 20.404 | 0.502 | 2.485 | -7.171 – 12.142 | 0.608 |

^a^ Model is adjusted for differences of NDI at baseline

^b^ Model is additionally adjusted for sex and age

CI = Confidence Interval

**VAS arm**

|  | **B^a^** | **(95% CI)^a^** | ***p*^a^** | **B^b^** | **95% CI^b^** | ***p*^b^** |
| --- | --- | --- | --- | --- | --- | --- |
| W52 | 6.838 | -9.774 – 23.451 | 0.413 | 8.749 | -8.401 – 25.898 | 0.346 |
| W104 | -9.015 | -26.773 – 8.742 | 0.314 | -9.603 | -27.796 – 8.589 | 0.295 |
| W260 | -22.583 | -51.377 – 6.210 | 0.121 | -26.570 | -53.183 – -0.044 | 0.050 |

^a^ Model is adjusted for differences of VAS arm pain at baseline

^b^ Model is additionally adjusted for sex and age

CI = Confidence Interval

**VAS neck**

|  | **B^a^** | **(95% CI)^a^** | ***p*^a^** | **B^b^** | **(95% CI)^b^** | ***p*^b^** |
| --- | --- | --- | --- | --- | --- | --- |
| W52 | 0.328 | -10.534 – 16.198 | 0.672 | 4.197 | -9.530 – 17.925 | 0.542 |
| W104 | -0.600 | -15.145 – 15.801 | 0.966 | -1.200 | -16.977 – 14.578 | 0.879 |
| W260 | -9.493 | -38.517 – 19.531 | 0.513 | -15.026 | -41.168 – 11.116 | 0.252 |

^a^ Model is adjusted for differences of VAS neck pain at baseline

^b^ Model is additionally adjusted for sex and age

CI = Confidence Interval
