# Supplementary material for: Association between fusion and clinical outcomes after anterior cervical discectomy at 1-, 2- and 5-year follow-up
Source: PLoS One. 2025 Dec 15;20(12):e0337909. doi: 10.1371/journal.pone.0337909 (PMC12704901; doi:10.1371/journal.pone.0337909)
Supplement: S3 Appendix — (DOCX) [file pone.0337909.s003.docx]

**Supporting information**

**S3 Appendix.** Clinical and radiological outcomes for each intervertebral device at 260 weeks FU.

| **Variable** | **ACD** | **ACDF** | **ACDA** | **P-value** |
| --- | --- | --- | --- | --- |
| Fusion | 23/24 (95.8%) | 20/23 (80%) | 13/20 (52%) | 0.020 |
| NDI | 25.2 ± 21.1 | 15.6 ± 15.3 | 13.4 ± 13.1 | 0.042 |
| VAS arm | 27.1 ± 31.8 | 16.0 ± 24.2 | 12.6 ± 19.2 | 0.130 |
| VAS neck | 32.0 ± 31.5 | 22.4 ± 25.0 | 14.7 ± 23.4 | 0.089 |

ACDA = anterior cervical discectomy with arthroplasty, ACDF = anterior cervical discectomy and fusion, ACD = anterior cervical discectomy without an intervertebral device, NDI = neck disability index, VAS = visual analog scale.
